# Advancing multimorbidity management in primary care: a narrative review

**DOI:** 10.1017/S1463423622000238

**Published:** 2022-07-01

**Authors:** Chanchanok Aramrat, Yanee Choksomngam, Wichuda Jiraporncharoen, Nutchar Wiwatkunupakarn, Kanokporn Pinyopornpanish, Poppy Alice Carson Mallinson, Sanjay Kinra, Chaisiri Angkurawaranon

**Affiliations:** 1 Department of Family Medicine, Faculty of Medicine, Chiang Mai University, Chiang Mai, Thailand; 2 Department of Non-communicable Disease Epidemiology, Faculty of Epidemiology and Population Health, London School of Hygiene and Tropical Medicine, London, UK; 3 Global Health and Chronic Conditions Research Group, Chiang Mai University, Chiang Mai Thailand

**Keywords:** chronic disease, guidelines, multimorbidity, primary care

## Abstract

**Background:**

Multimorbidity, defined as the coexistence of two or more chronic conditions in the same individual, is becoming a crucial health issue in primary care. Patients with multimorbidity utilize health care at a higher rate and have higher mortality rates and poorer quality of life compared to patients with single diseases.

**Aims:**

To explore evidence on how to advance multimorbidity management, with a focus on primary care. Primary care is where a large number of patients with multimorbidity are managed and is considered to be a gatekeeper in many health systems.

**Methods:**

A narrative review was conducted using four major electronic databases consisting of PubMed, Cochrane, World Health Organization database, and Google scholar. In the first round of reviews, priority was given to review papers summarizing the current issues and challenges in the management of multimorbidity. Thematic analysis using an inductive approach was used to build a framework on how to advance management. The second round of review focused on original articles providing evidence within the primary care context.

**Results:**

The review found that advancing multimorbidity management in primary care requires a health system approach and a patient-centered approach. The health systems approach includes three major areas: (i) improves access to care, (ii) promotes generalism, and (iii) provides a decision support system. For the patient-centered approach, four key aspects are essential for multimorbidity management: (i) promoting doctor-patient relationship, (ii) prioritizing health problems and sharing decision-making, (iii) supporting self-management, and (iv) integrating care.

Advancement of multimorbidity management in primary care requires integrating concepts of multimorbidity management guidelines with concepts of patient-centered and chronic care models. This simple integration provides an overarching framework for advancing the health care system, connecting the processes of individualized care plans, and integrating care with other providers, family members, and the community.

## Background

Multimorbidity, defined as the coexistence of two or more chronic conditions in the same individual (World Health Organization, 2016), is becoming a crucial health issue in primary care. In the past two decades, the prevalence of chronic diseases has doubled, and the proportion of patients with four or more chronic diseases has increased by approximately 300% (Uijen and van de Lisdonk, 2008). However, in contrast, many current health services, models of care, and clinical practice guidelines (CPGs) are usually based on a single disease approach(World Health Organization, 2016), which may not be appropriate for patients with multimorbidity (van Oostrom *et al.*, 2014). Caring for patients with multimorbidity requires a reorientation in the health system as they are high utilizers of health care resources (van den Bussche *et al.*, 2011, van Oostrom *et al.*, 2014, Bähler *et al.*, 2015). Besides, clinical practice in primary care needs to adapt from the control of specific diseases to more holistic measures such as functional status and quality of life (Wallace *et al.*, 2015, Kernick *et al.*, 2017).

### Challenges in multimorbidity management in primary care: the ‘interactions’

Evidence suggests that patients with multimorbidity have higher mortality rates (Di Angelantonio *et al.*, 2015, Nunes *et al.*, 2016, Willadsen *et al.*, 2018) and poorer quality of life (Fortin *et al.*, 2004, Marengoni *et al.*, 2011, Kanesarajah *et al.*, 2018) compared to patients with single diseases. The difficulty occurs because the health services and CPGs usually focus on only a single disease (Kernick *et al.*, 2017). The task of following and incorporating multiple disease-specific guidelines is one of the key complexities of management, potentially leading to inappropriate, burdensome treatment plans. The complexity in incorporating many disease treatment guidelines can be referred to as ‘interaction’. Usually mentioned in terms of interactions between multiple medications (drugs), interactions can more broadly be categorized into three major groups: disease-disease, disease-treatment, and treatment-treatment (Muth *et al.*, 2014a; 2014b). The word treatment is used instead of the drug to include non-pharmacological treatment, such as exercise and dietary management. A recent study trying to identify disease-treatment and treatment-treatment serious interactions from 12 different National Institute for Health and Care Excellence (NICE) CPGs with three index conditions (type 2 diabetes, heart failure, and depression) identified 48 possible serious disease-treatment interactions and 333 potential drug-drug interactions (Dumbreck *et al.*, 2015). The details of the three types of potential interactions between diseases and treatments are highlighted below.

#### Disease-disease interaction

Diseases can interact in many ways. Having multiple diseases could result in difficulty in evaluating symptoms interfering with the typical clinical presentation or laboratory interpretation, complicating the differential diagnosis. More than one disease can contribute to one non-specific symptom or health event. This is well illustrated in older adults, especially in the frail population. Geriatric syndromes, a group of clinical signs or symptoms, occur from multiple etiologies and pathologies, which lead to difficulty in diagnosis and treatment (Mitty, 2010). Also, one disease can be a precipitating or a predisposing factor to another, or disease pathology can be shared, which might worsen the patient’s condition. This is usually found in diseases involving cardiovascular risks, such as diabetes mellitus, dyslipidemia, hypertension, and cardiovascular events. Thus, the progression of one disease can accelerate other diseases’ progression or lead to other conditions (Prados-Torres *et al.*, 2012). Also, disease-disease interaction is not only found between physical health problems. Interaction with mental health disorders has been frequently reported (Cohen, 2017). For example, cardiovascular diseases may trigger, or mechanisms of disease progression may also contribute to mental disorders. On the other hand, stress and depression can trigger disease exacerbation or contribute to disease development (Stein *et al.*, 2019). A study extracting data from a medical database in Scotland found more than one-third of people with multimorbidity have at least one mental health issue (Barnett *et al.*, 2012).

#### Disease-treatment interaction

More diseases lead to more prescriptions and an increased risk of interactions with treatment. Drugs recommended for one condition could be contraindicated in another or should be avoided, and treatment might mask or alter the sign of some conditions. For example, in a patient with hypertension and diabetes, the beta-blocker prescribed for the treatment of hypertension may mask the initial sign of hypoglycemic symptoms. Some disease-treatment interactions could worsen a patient’s condition, such as sedative/hypnotic/anticholinergic drugs for the cognitively impaired patient. Other disease-treatment interactions may cause new symptoms from an adverse drug event. Unawareness of the possible side effects and interactions from prescribed medication, physicians may prescribe more medication for these symptoms. This is called a prescription cascade (Rochon and Gurwitz, 2017), which is the process of prescribing a new medication to treat a side effect from another medication, which does not provide a true benefit to the patient.

Moreover, non-pharmacological treatment options for one disease, such as exercise or dietary control, must also be carefully considered when dealing with multimorbidities. For example, exercise and physical activity is an important component in controlling most chronic conditions (de Souto Barreto, 2017); however, it should be prescribed to patients based on their overall conditions. Options for patients with poorly controlled diabetes with osteoarthritis of the knee or chronic lung conditions should be different from the patient with well-controlled diseases with no physical limitations.

There is also some evidence of positive interactions between disease-treatment, which can be classified as a synergistic treatment effect (Muth *et al.*, 2014a). For example, some medications could be used appropriately or effectively for treating more than one condition, such as alpha-blockers for controlling symptoms of benign prostatic hypertension and treating high blood pressure. On the other hand, some medications are not recommended for both conditions, such as some NSAIDs in a patient with chronic kidney disease and heart disease.

#### Treatment-treatment interaction

Multiple medications, sometimes referred to as polypharmacy, can increase the chance of drug-drug interaction (Marengoni and Onder, 2015, Molokhia and Majeed, 2017). Potentially serious interactions between drugs recommended by clinical guidelines are common (Dumbreck *et al.*, 2015). This can change the therapeutic effects of the medication or can cause adverse effects and lead to undesirable outcomes. Moreover, complex regimens – multiple dosing and time – could reduce medication adherence (Ingersoll and Cohen, 2008). Some non-pharmacologic treatments can interrupt compliance to medication prescription. For example, in end-stage kidney disease, patients have to restrict their fluid intake while they usually have several medications to take orally with water for the comorbid conditions. Another example is a lifestyle modification prescription for a frail older adult patient who has malnutrition with uncontrolled diabetes and dyslipidemia. It is important to improve his/her nutrition by increasing caloric intake. However, if he/she is too strict to the specific disease guidelines for those two latter conditions improving their nutrition is nearly impossible. Therefore, the doctor should balance the treatment plan, maximizing benefit from existing treatments and stopping the treatments with limited benefits (Kernick *et al.*, 2017).

Given the complexities of the three types of interactions in multimorbidity management described above, the narrative review sought to explore evidence on how to advance multimorbidity management, with a focus on primary care where a large number of patients with multimorbidity are managed and are considered to be the gatekeepers in many health systems (Cassell *et al.*, 2018).

## Methods

### Search strategy

We conducted a narrative review (Ferrari, 2015) of the literature using four major electronic databases consisting of PubMed, Cochrane, World Health Organization database, and Google scholar using variations of the following keyword search and MeSH terms

Search terms:

1. (multimorbid*) AND (Primary care) + filter: human, English
2. (multimorbid* OR (multiple chronic diseases)) AND (Primary care) AND (Guide* OR recommend*) + filter: human, English
3. (multimorbid* OR (multiple diseases)) AND (multidisciplinary* OR interprofessional*) AND (primary care) + filters: human, English
4. (multimorbid* OR (multiple diseases)) AND (family OR career OR (caregiver)) AND (primary care) + filters: human, English
5. (multimorbid* OR (multiple diseases)) AND (community OR public OR social) AND (primary care) + filters: human, English

### Article selection and data synthesis

**Figure 1.**
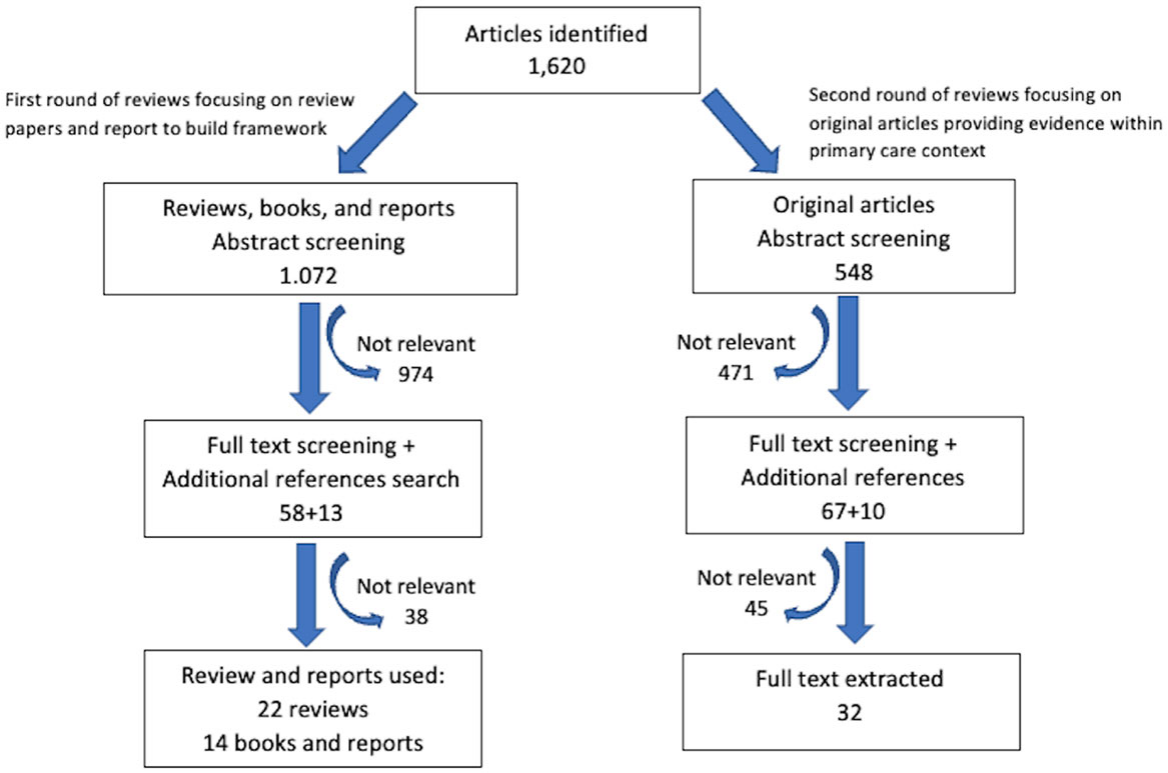
Flow diagram of articles

Only articles published in English were reviewed. In the first round of reviews, priority was given to review papers (including systematic reviews, narrative reviews, and reports) summarizing the current issues and challenges in the management of multimorbidity. Thematic analysis using an inductive approach was used to build a framework on how to advance management (Castleberry and Nolen, 2018). This was done by creating key themes and subthemes based on the information gathered from the first round of reviews. The second round of reviews focused on original articles providing evidence within the primary care context (Figure 1). There were no exclusion criteria on the type of study design used. We included intervention studies, epidemiological studies, and implementation studies. The reference lists of all review articles were searched for additional studies. The evidence found from the second round of review based on the original articles was also used to reiterate the proposed framework obtained from the thematic analysis of review papers. A summary of the 32 original articles used as evidence to support the framework proposed in the review is included in Supplementary Table 1 and Supplementary Table 2.

Articles were screened and reviewed by four authors; all are practicing family physicians with experience in multimorbidity management. Each article is screened by at least two reviewers. If there is a discrepancy, it was resolved by consensus in consultation with the senior author, who is a family physician with postgraduate training in population health and over ten years of experience in public health. The thematic analysis was led by two co-authors with experience in qualitative research.

## Results

**Figure 2.**
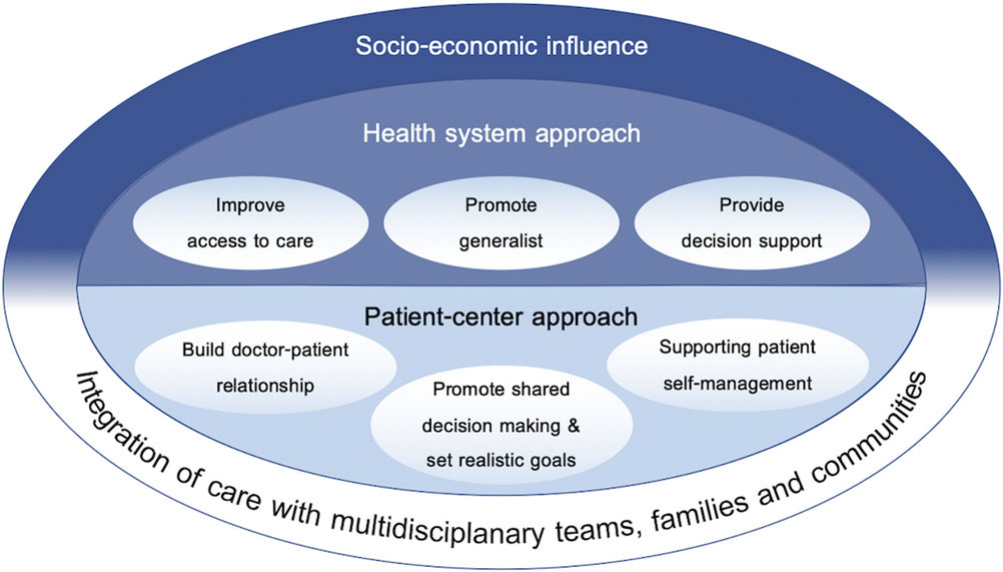
Framework for advancing multimorbidity management in primary care

Based on our searches, evidence suggests that advancing multimorbidity management in primary care requires both a health system-based approach to reorientate health services and a more patient-centered approached for providers to support the complexities of care for patients with multimorbidity is needed (Figure 2).

### Advancing multimorbidity management through health system-based approach

It is important to note that improving the health system for better patient care and care delivery may not cover all aspects of multimorbidity management. Many populations are at higher risk and are more vulnerable to the impacts of multimorbidities, such as those with lower socioeconomic status (Barnett *et al.*, 2012, Pathirana and Jackson, 2018, Andrew Wister *et al.*, 2020), underlying the need to address the social determinants of health. Nevertheless, to advance multimorbidity management in primary care, the literature suggests three major areas that need to be addressed: i) improve access to care, ii) promote generalism, and iii) provide a decision support system. Addressing these three areas should help reduce the fragments of services and responsibility and drive the medical system to provide accountable, accessible, comprehensive, and coordinated care for those with multimorbidity.

#### Improve access to care

Multimorbid patients require more medical attention resulting in a higher rate of health care contact compared to the non-multimorbid population (Bähler *et al.*, 2015). Some patients are unable to maintain regular health care visits due to barriers in access to care (Moffat and Mercer, 2015, Wallace *et al.*, 2015). Evidence identified two major barriers in accessing health care in multimorbid patients: i) the ability to pay and ii) the ability to reach necessary health services (World Health Organization, 2016, Foo *et al.*, 2020)

The ‘ability to pay’ can be dealt with by providing access to universal health coverage. Appropriated universal health coverage has been shown to improve access to care and improve health outcomes (World Health Organization, 2013). The World Health Organization also suggests universal health coverage as one of the important steps toward improving care in multimorbidity (World Health Organization, 2016). This can be done briefly by giving a high priority to achieving full population coverage of an affordable package of services through publicly governed, mandatory financing mechanisms and sustained political commitment from the highest level of government (David Nicholson *et al.*, 2015).

The ‘ability to reach’ can potentially be dealt with by allocating primary care sites closer to the community (Glass *et al.*, 2017). While evidence specifically assessing the multimorbid population and access to primary care is lacking, existing evidence suggests that better access to primary care has proven successful in improving health outcomes for many different conditions (Kravet *et al.*, 2008, Bynum *et al.*, 2011, Shi, 2012). Also, improving geographical reach alone may not be able to handle the complex needs of multimorbid patients. Necessary health services must also be presented at the working sites. A potential way to improve reach to necessary health services is to provide a policy that emphasizes health resource allocation and systems for consultation and referrals (Hsieh *et al.*, 2015). In addition, due to the COVID pandemic, telemedicine and telehealth have been used to help improve access to care (North, 2020, Sinsky, 2020). While a conceptual model for telehealth and chronic disease management has been proposed (Salisbury *et al.*, 2015), limited evidence has been published on specifically assessing its impact on the multimorbid population. A quasi-experimental study from Spain suggested that the use of telemedicine in primary care can help improve health outcomes, such as better disease control and reduce emergency hospitalization among those with chronic conditions (Orozco-Beltran *et al.*, 2017). A recent review has summarized that while the telehealth approach has been proven successful for the management of common chronic diseases, there is high heterogeneity in the technology use, and further clinical studies are needed to provide robust evidence on clinical efficacy and safety (Omboni *et al.*, 2020).

#### Promote generalism

The complexity of multimorbidity often requires some degree of coordination between different specialists and often leads to fragmentation and disruption of care (Moffat and Mercer, 2015, Wallace *et al.*, 2015). Fragmented care results in poorer care quality, poor resource utilization, and a higher hospitalization rate of ambulatory care sensitive conditions – conditions that, ideally, primary care should be able to prevent (Frandsen *et al.*, 2015). Impacts of disruption of care include poorer clinical outcomes, such as increased hospitalizations and mortality rates and higher treatment costs.

Generalism is a concept of seeing a person as a whole and providing broad and holistic health care deliveries that are relevant to the patient’s problems (Howe, 2012). The concept itself aims to better understand patients’ disease-illness and context, widen the spectrum of care, improve coordination of care, reduce fragmentation of care, and improve continuity of care. Generalism would also reduce care disruption and improve multimorbidity management (May *et al.*, 2009, World Health Organization, 2016, Vishal Ahuja and Staats, 2020).

Promoting generalism within the health care system can be done in several ways. Evidence suggests that generalists and family physicians are trained to provide medical care comprehensively and holistically (World Health Organization, 2016), which is a key component in promoting generalism (2011). Also, training health workers to provide comprehensive medical services with nurse managers or care managers (Suriyawongpaisal *et al.*, 2019) while adjusting the health delivery system to promote task sharing and task shifting between providers in primary care could help improve the continuity of care while reducing fragmentation and disruption of care among patients with multimorbidity.

#### Provide decision support

CPG is one form of decision support that guides patient care. However, the methodology for developing CPGs is usually based on evidence or research studies that focus on specific diseases (Tinetti *et al.*, 2004, World Health Organization, 2016). This causes concern about how CPGs should be applied to patients with multiple comorbidities. A couple of studies that tried applying CPGs in hypothetical multimorbid patients resulted in an excessive number of drugs and lifestyle modifying prescriptions. Such practice could cause a burden to the patients and introduce risks of unexpected adverse health effects from drug interactions, resulting in the patient’s poor treatment adherence and poor treatment outcomes. (Boyd *et al.*, 2005, Okeowo *et al.*, 2018). The evidence points out a need to create CPGs for patients with multiple comorbidities. A framework for the development of CPGs that consider multimorbidity has been developed (Uhlig *et al.*, 2014), but CPGs regarding how to provide particular treatments given a particular set of multiple comorbidities are still limited (World Health Organization, 2016). From our review, to date, BMJ Best Practice is one of the few sources for CPGs that incorporate the concept of multimorbid (BMJ Best Practice). However, the online guideline tools, first launched in September 2020, are still limited to common acute conditions such as COVID-19 and acute exacerbations of chronic obstructive pulmonary disease and require an access fee.

Since the early 2000, with the advancement in computer technology, CPGs have been formatted as computer-interactable guidelines (CIGs). This enables the development of CIG-driven clinical decision support systems (CDSSs). Many CIG-based CDSSs have been developed to detect interactions between CIGs to support physician’s care delivery. However, there is still a huge gap until they are applicable in an actual clinical setting as they are not easily adaptable, generalizable, or re-usable in their current state and still have many limitations in real-life situations (Bilici *et al.*, 2018).

Many CDSSs have been developed to detect potential adverse drug reactions in a patient with multiple drug prescriptions from the electronic medical record (EMR). A study regarding the effectiveness of EMR-enabled CDSSs in specifically multimorbid populations is lacking. EMR-enabled CDSSs were proven to be most effective in reducing potentially inappropriate medications in the hospital setting, less effective in an ambulatory care setting, and borderline effective in residential aged care facilities (Scott *et al.*, 2018). The population in the studies is not limited to the multimorbid patient but mostly are elderly with a high prevalence of multimorbidity. The results might be applicable to the multimorbid population.

### Advancing multimorbidity management through a patient-centered approach

In addition to advancing health systems factors to promote multimorbidity management, for providers directly involved in patient care, evidence suggests that the core principles of patient-centered medicine (PCM) (Muth *et al.*, 2014b, Wallace *et al.*, 2015) and the chronic care model (CCM) (Boehmer *et al.*, 2018) are crucial towards advancing care for a patient with multimorbidity. The following PCM and CCM components should be embedded in multimorbidity management in primary care

#### Building and maintaining a good doctor-patient relationship as a partnership

Primary care providers usually prioritize having a doctor-patient relationship as an important factor in successful treatment outcomes (Damarell *et al.*, 2020). A good partnership and trust will help towards agreement or the willingness of patients to follow the care plan and increase the chance of better health outcomes (McGilton *et al.*, 2018)

To build and maintain a good doctor-patient relationship, the primary care provider needs to understand the patient as a whole person (psychological, social, and spiritual aspects). The ability to provide for a patient over a long period of time is beneficial for knowing the history of patients’ diseases and illness experience, the life context of a career, specific life circumstances, and spiritual aspects (Damarell *et al.*, 2020). Asking and listening to patient/family concerns not only show that the provider care for the patient and build a partnership (Poitras *et al.*, 2018) but also could provide insights into an aspect of the patient’s personal and social circumstance which might impact the therapeutic acceptance and success (Damarell *et al.*, 2020). Knowing patients’ resources and limitations assists the provider to create an individualized care plan for each patient’s unique circumstances and illness experience (Bogner and de Vries, 2008, Poitras *et al.*, 2018) which is essential for patients with multimorbidity (Bogner and de Vries, 2008, McGilton *et al.*, 2018).

#### Prioritization of health problems, promoting shared decision-making, and setting realistic goals

Based on the potential interactions and disease trajectories, prioritization of health problems in a patient with multimorbidity needs to take into account the patient’s concerns, values, goals, and preferences along with the multiple clinical and disease-specific interactions and risk factors. To help understand and prioritize these complex issues and problems that patients with multimorbid may have, simple questions such as ‘What is bothering you most?’ or ‘What would you like to focus on today?’ may be used to help elicit first responses (Damarell *et al.*, 2020).

Involving patients in the decision-making process results in better outcomes such as increased patient satisfaction, better adherence to treatment regimens, improved functional status, and optimized self-management (McGilton *et al.*, 2018; Rijken *et al.*, 2017). With multimorbidity problems, a longer consultation time is needed. Thus, using available shared decision-making tools may help support the process (Damarell *et al.*, 2020). An example of such a decision-making tool is the three-step talk: step 1) ‘*choice talk’,* making sure that patients know that reasonable options are available, step 2) ‘*option talk’,* providing more detailed information about options, and step 3) ‘*decision talk’*, supporting the work of considering preferences and deciding what is best and effective in primary care (Wallace *et al.*, 2015).

A thorough interaction assessment of the patient’s conditions, treatments, consultation, and context can lead to multiple treatment goals, which may include targeting symptoms, functional ability, quality of life, desired patient outcomes, etc. (Palmer *et al.*, 2018). For individualized care planning, the provider should find agreement with the patient and/or family members (with the patient’s permission for members to be involved) on the responsibility for coordination of care, agreement of goal and timing of follow-up, and how to access urgent care and arrangement for more frequent follow-up as needed especially for those with complex disease management (Kernick *et al.*, 2017). Furthermore, in each follow-up consultation, the individualized care plans should be reviewed and modified at each reassessment (Palmer *et al.*, 2018).

#### Supporting patient self-management

For a patient with multimorbidity, promoting self-management is a key factor because patients often have numerous conditions to monitor simultaneously, many of which affect the other comorbidities. Also, the intervention of care and treatment usually requires lifestyle changes. Therefore, active involvement of the patient is crucial to achieving expected health outcomes (Palmer *et al.*, 2018, Poitras *et al.*, 2018). Several key success factors that can promote self-management include using the participatory approach, understanding the patient’s situation, enhancing the patient’s motivation, reinforcing adherence, providing educational resources and skills, and developing peer support through group meetings (Poitras *et al.*, 2018). The evidence shows that self-management education programs can help patients with single chronic diseases as well as those with multimorbidity (Lynch *et al.*, 2014). Additional patient self-management support materials should be provided, such as CDs, videos, booklet, or other written material, and self-monitoring devices appropriate to their condition (Katon *et al.*, 2010, Mercer *et al.*, 2016, Angkurawaranon *et al.*, 2020). For example, the CARE Plus intervention, which is a whole-system primary area-based complex intervention, provided mindfulness-based stress management CDs and a cognitive-behavioral therapy-derived self-help booklet about the intervention (Mercer *et al.*, 2016). In a self-monitoring program for a patient with depression and diabetes or coronary heart disease, patients received blood pressure or blood glucose meters (Katon *et al.*, 2010).

#### Integration of care with multidisciplinary teams, families, and communities

Integration is a group of methods and models designed to promote connectivity and reduce boundaries between ‘cure’ and ‘care’ sectors (Kodner, 2002). Integrated care is likely needed to improve quality of care and quality of life, especially in people with complex care needs (Leutz, 1999). It is defined as the search to connect the health care system with another human service (Leutz, 1999). For patients with multimorbidity, integration with a multidisciplinary team and their family/community is essential to advance multimorbidity management

Multidisciplinary working is the cooperation across service providers by conjugating knowledge, skills, and best practice to explore extraordinary problems and reach the best solution for patients with complex care needs (Susan Swientozielskyj *et al.*, 2014). To care for patients with multimorbidity, a collaboration between physicians, nurse case managers can play an essential role in the area of providing a central and continuous point of contact, as well as promoting patient’s self-health management (eg, person-centered assessment, assisting the patient to set the goal of care, enhancing patient and caregiver education, delivering preventive care, monitoring patient’s status, together with providing patient’s partnership) (Sommers *et al.*, 2000, Hogg *et al.*, 2008, Katon *et al.*, 2010, Boult *et al.*, 2011). Examples of successful programs include the participation of psychiatrists to help provide psychological support, pharmacological treatment, and mental monitoring for mental health support (Barley *et al.*, 2014), having a nurse or social worker as a case manager to evaluate the patient in the home (Sommers *et al.*, 2000). Several studies have shown that the collaboration of pharmacists in the role of medication reviewer and assisted care plan manager in the primary care team benefits the outcomes of disease control, preventive care, and medication safety (Krska *et al.*, 2001, Hogg *et al.*, 2009, Howard-Thompson *et al.*, 2013, Köberlein-Neu *et al.*, 2016). Moreover, co-working with health educators, dietitians, home-care specialists, and social workers has also shown to be an effective disease control strategy in patients with multiple chronic conditions (Bogner and de Vries, 2008, Lynch *et al.*, 2014, Köberlein-Neu *et al.*, 2016).

Families and communities of patients may also need to be involved to reach the best health care (World Health Organization, 2016). Family support is one of the essential resources for a patient with multimorbidity. Family members or caregivers help navigate the health care system to obtain services (Zulman *et al.*, 2015). A qualitative study revealed that family caregivers can fill the gaps in the fragmented medical system. They play multiple roles, including coordinating care across transitions, accessing and coordinating medical care services, communicating with physicians and services, and providing information regarding patients’ medical history (Levine *et al.*, 2003–2004, Bookman, 2007). Family caregivers can also assist in motivating patients to make behavioral changes (Naganathan *et al.*, 2016) and share in medical decision-making (Kernick *et al.*, 2017).

Community-based integrated care is the combined terms of community-based care and integrated care. Community-based care is a health system that is designed and driven by community health needs, beliefs, and values which promotes engagement and compliance of communities that are driven by their system. Since integrated care mainly focuses on the reduction of fragmentation in health care delivery, community-based integrated care provides an outlook on the way the various rationalization strategies could be combined by taking the reduction of fragmentation in health care delivery and a consistent focus on the health of the community as the starting point (Plochg and Klazinga, 2002). Older adults with multimorbidity may face disability, functional impairment, and chronic disease burden. Access to care is now challenged by environmental factors and social determinants of health. For example, the Richmond Health and Wellness Program is a collaboration of health professionals from schools of nursing, pharmacy, medicine, social work, allied health, and psychology which was initiated to develop the strategy to reduce barriers to access to care. This community-based partnership was contributed by the cocreation of the program with residents, students, providers, community agencies, and institutional leaders. By having support in shaping policy, securing grants, and offsetting negative social determinants of health, these partnerships bring positive outcomes (Parsons *et al.*, 2019).

Based on the framework and evidence mentioned in this review, a simple checklist has been made to help summarize the key assessments that should help primary care providers manage patients with multimorbidity. (Table 1 Simple Multimorbidity Assessment Checklist for Primary Care **–** SMAC)

**Table 1.** Simple Multimorbidity Assessment Checklist for primary care

No.	Items	Check
1.	Assessment of the patient	
	1.1 Physical symptoms	
	1.2 Psychological status	
	1.3 Functional status	
2.	Review all diseases/conditions	
	2.1 Disease trajectory	
	2.2 Identified risk	
3.	Review of all treatments	
	3.1 Non-pharmacological treatment, such as exercise or dietary control	
	3.2 Pharmacological treatment, including OTCs and herbal, complementary, and alternative medicine	
4.	Review clinical practice guidelines and assess interactions	
	4.1 Disease-disease interaction	
	4.2 Disease-treatment interaction	
	4.3 Treatment-treatment interaction	
5.	Understanding patient context and concerns	
	5.1 Patient’s background, such as career, marital status, family/friend, and spiritual beliefs	
	5.2 The patient’s perspective of diseases, including idea, feeling, function, and expectations	
6.	Finding common ground	
	6.1 Identifying the patient’s goal	
	6.2 Prioritize the problems (considering the importance and urgency of conditions and also patient preference)	
	6.3 Informed decision-making: choice talk, options talk, decision talk	
7.	Set individual care plan	
	7.1 Setting realistic treatment goals and care plan	
	7.2 Encouraging self-management	
	7.3 Resources, such as multidisciplinary teams, families, communities, and support system	
8.	Continuity of care and follow-up visits	
	8.1 Schedule follow-up	
	8.2 Review patient goals, such as behavior and clinical outcome, and provide support as needed	

## Discussion

The review found that the literature on the implementation of programs for advancing multimorbidity management within primary care is still relatively scarce and is mostly from developed countries (Wallace *et al.*, 2015, Rijken *et al.*, 2018). As evident in the review process and this review, many published literature (Harris *et al.*, 2013; Wallace *et al.*, 2015; Rijken *et al.*, 2018) and agencies, such as the European Union (Rijken *et al.,* 2017) and NICE (Kernick *et al.*, 2017), have published guidelines on multimorbidity management. While many aspects between the reports and our review may overlap, the scope of this narrative review was to provide a framework and evidence on how to advance multimorbidity management with a particular focus on primary care, thus integrating multimorbidity management guidelines with concepts of PCM and the CCM. This simple integration provides an overarching framework for advancing the health care system by connecting to the processes of individualized care plans and integration of care with other providers, family members, and the community (Figure 2).

It is also important to note that multiple issues and difficulties come with implementing frameworks to advance multimorbidity management (Damarell *et al.*, 2020). In Europe, getting all the care packages required for the management of multimorbidity into a basic insurance package has faced difficulties (Rijken *et al.*, 2017). In Thailand, finding sustainable sources of financing and training primary care teams is a major concern as the sustainability of primary care development relies mainly on partnerships, international financial support, and expertise from overseas (Suriyawongpaisal *et al.*, 2019). A lack of computer skills among care professionals and patients, inadequate ICT infrastructure, and inadequate funding for structural implementation and innovation in supportive eHealth tools are also important barriers when implementing electronic decision support systems (Rijken *et al.*, 2017, Dornan *et al.*, 2019). Traditional norms, values, and work processes can become a barrier to the implementation of patient-centered integrated care due to a lack of managerial vision of patient centeredness in care organizations (Wallace *et al.*, 2015; Rijken *et al.*, 2017). For family members and/or caregivers who have a responsibility in caring for patients with multimorbidity, literature has documented that some have trouble with accessing helpful information, assessing the quality of services, understanding what information is necessary to get services, and anticipating what will be needed (Bookman, 2007). As these difficulties are documented, more evidence is needed to share the lessons learned and how to overcome such barriers to advance multimorbidity management in primary care.

With the knowledge of multimorbidity management in primary care in its infancy, especially for developing countries, we proposed a number of pressing research questions cross-cutting these themes in both clinical science and implementation sciences perspectives. These questions include: How can we improve evidence base for management of patients with multimorbidity while incorporating patient perspectives? What role(s) can para-health professionals play in scaling up and scaling out (reach) of management of multimorbidity? What training and education for (para) health professionals are needed to advance multimorbidity care toward a patient-centered approach? What sustainable funding models are needed to deliver improvements in multimorbidity care? How can technology-based platforms be integrated into health systems at scale to support clinical decision-making and long-term management of multiple chronic conditions?

### Conclusion

The review provides a framework and evidence to support a framework for advancing multimorbidity management in primary care. Advancing multimorbidity management in primary care requires both a health system approach to help improve the access, delivery, and quality of care as well as a patient-centered approach so that necessary components of PCM and the CCM are incorporated into the management of patients with such complex conditions. Based on the review, a Simple Multimorbidity Assessment Checklist for Primary Care has also been proposed to help guide providers provide management for those with multimorbidity. However, as it has not been validated, future studies on its clinical usefulness are required. Finally, with multimorbidity management in its infancy, especially for most developing countries, we have proposed a number of pressing research questions cross-cutting these themes.

## Supplementary material

For supplementary material accompanying this paper visit http://doi.org/10.1017/S1463423622000238. click here to view supplementary material

## References

[ref] Andrew Wister LR , Shashank A , Byron Walker B and Schuurman N (2020) Multimorbidity and socioeconomic deprivation among older adults: a cross-sectional analysis in five Canadian cities using the CLSA. Journal of Aging and Environment 16, 127–131.

[ref2] Angkurawaranon C , Papachristou Nadal I , Mallinson PAC , Pinyopornpanish K , Quansri O , Rerkasem K , Srivanichakorn S , Techakehakij W , Wichit N , Pateekhum C , Hashmi AH , Hanson K , Khunti K and Kinra S (2020) Scalable solution for delivery of diabetes self-management education in Thailand (DSME-T): a cluster randomised trial study protocol. BMJ Open 10, e036963. 10.1136/bmjopen-2020-036963 PMC7537447 33020090

[ref3] Bähler C , Huber CA , Brüngger B and Reich O (2015) Multimorbidity, health care utilization and costs in an elderly community-dwelling population: a claims data based observational study. BMC Health Services Research 15, 23. 25609174 10.1186/s12913-015-0698-2 PMC4307623

[ref4] Barley EA , Walters P , Haddad M , Phillips R , Achilla E , McCrone P , Van Marwijk H , Mann A and Tylee A (2014) The UPBEAT nurse-delivered personalized care intervention for people with coronary heart disease who report current chest pain and depression: a randomised controlled pilot study. PLoS One 9, e98704. 24901956 10.1371/journal.pone.0098704 PMC4047012

[ref5] Barnett K , Mercer SW , Norbury M , Watt G , Wyke S and Guthrie B (2012) Epidemiology of multimorbidity and implications for health care, research, and medical education: a cross-sectional study. Lancet 380, 37–43. 22579043 10.1016/S0140-6736(12)60240-2

[ref6] Bilici E , Despotou G and Arvanitis TN (2018) The use of computer-interpretable clinical guidelines to manage care complexities of patients with multimorbid conditions: a review. Digit Health 4, 2055207618804927. 30302270 10.1177/2055207618804927 PMC6172935

[ref7] BMJ Best Practice. Introducing the comorbidities tool from BMJ best practice [Online]. Available: https://bestpractice.bmj.com/info/introducing-the-comorbidities-tool-from-bmj-best-practice/ [Accessed 23 March 2020].

[ref8] Boehmer KR , Abu Dabrh AM , Gionfriddo MR , Erwin P and Montori VM (2018) Does the chronic care model meet the emerging needs of people living with multimorbidity? A systematic review and thematic synthesis. PLoS One 13, e0190852. 29420543 10.1371/journal.pone.0190852 PMC5805171

[ref9] Bogner HR and de Vries HF (2008) Integration of depression and hypertension treatment: a pilot, randomized controlled trial. The Annals of Family Medicine 6, 295–301. 18626028 10.1370/afm.843 PMC2478504

[ref10] Bookman AHM (2007) Family caregivers: a shadow workforce in the geriatric health care system? Journal of Health Politics, Policy and Law 23, 1005–1041. 10.1215/03616878-2007-040 18000158

[ref11] Boult C , Reider L , Leff B , Frick KD , Boyd CM , Wolff JL , Frey K , Karm L , Wegener ST , Mroz T and Scharfstein DO (2011) The effect of guided care teams on the use of health services: results from a cluster-randomized controlled trial. Archives of Internal Medicine 171, 460–466. 21403043 10.1001/archinternmed.2010.540 PMC4450357

[ref12] Boyd CM , Darer J , Boult C , Fried LP , Boult L and Wu AW (2005) Clinical practice guidelines and quality of care for older patients with multiple comorbid diseases: implications for pay for performance. JAMA 294, 716–724. 16091574 10.1001/jama.294.6.716

[ref13] Bynum JP , Andrews A , Sharp S , McCollough D and Wennberg JE (2011) Fewer hospitalizations result when primary care is highly integrated into a continuing care retirement community. Health Affairs (Millwood) 30, 975–984. 10.1377/hlthaff.2010.1102 PMC4096231 21555482

[ref14] Cassell A , Edwards D , Harshfield A , Rhodes K , Brimicombe J , Payne R and Griffin S (2018) The epidemiology of multimorbidity in primary care: a retrospective cohort study. British Journal of General Practice 68, e245–e251. 10.3399/bjgp18X695465 PMC5863678 29530918

[ref15] Castleberry A and Nolen A (2018) Thematic analysis of qualitative research data: is it as easy as it sounds? Currents in Pharmacy Teaching and Learning 10, 807–815. 30025784 10.1016/j.cptl.2018.03.019

[ref16] Cohen A (2017) Addressing comorbidity between mental disorders and major noncommunicable diseases (2017). Denmark: World health Organization Regional Office for Europe.

[ref1] Commission on Generalism (2011) Guiding patients through complexity: modern medical generalism. London: Royal College of General Practitioners and the Health Foundation.

[ref17] Damarell RA , Morgan DD and Tieman JJ (2020) General practitioner strategies for managing patients with multimorbidity: a systematic review and thematic synthesis of qualitative research. BMC Primary Care 21, 131. 10.1186/s12875-020-01197-8 PMC7331183 32611391

[ref18] David Nicholson RY , Warburton W and Fontana G (2015) Delivering universal health coverage: a guide for policy makers. World Innovation Summit for Health. https://www.wish.org.qa/reports/delivering-universal-health-coverage-a-guide-for-policymakers/

[ref19] De Souto Barreto P (2017) Exercise for multimorbid patients in primary care: one prescription for all? Sports Medicine 47, 2143–2153. 28386651 10.1007/s40279-017-0725-z

[ref20] Di Angelantonio E , Kaptoge S , Wormser D , Willeit P , Butterworth AS , Bansal N , O’Keeffe LM , Gao P , Wood AM , Burgess S , Freitag DF , Pennells L , Peters SA , Hart CL , Håheim LL , Gillum RF , Nordestgaard BG , Psaty BM , Yeap BB , Knuiman MW , Nietert PJ , Kauhanen J , Salonen JT , Kuller LH , Simons LA , Van der Schouw YT , Barrett-Connor E , Selmer R , Crespo CJ , Rodriguez B , Verschuren WM , Salomaa V , Svärdsudd K , Van der Harst P , Björkelund C , Wilhelmsen L , Wallace RB , Brenner H , Amouyel P , Barr EL , Iso H , Onat A , Trevisan M , D’Agostino RB , Cooper C , Kavousi M , Welin L , Roussel R , Hu FB , Sato S , Davidson KW , Howard BV , Leening MJ , Leening M , Rosengren A , Dörr M , Deeg DJ , Kiechl S , Stehouwer CD , Nissinen A , Giampaoli S , Donfrancesco C , Kromhout D , Price JF , Peters A , Meade TW , Casiglia E , Lawlor DA , Gallacher J , Nagel D , Franco OH , Assmann G , Dagenais GR , Jukema JW , Sundström J , Woodward M , Brunner EJ , Khaw KT , Wareham NJ , Whitsel EA , Njølstad I , Hedblad B , Wassertheil-Smoller S , Engström G , Rosamond WD , Selvin E , Sattar N , Thompson SG and Danesh J (2015) Association of Cardiometabolic Multimorbidity With Mortality. JAMA 314, 52–60. 26151266 10.1001/jama.2015.7008 PMC4664176

[ref21] Dornan L , Pinyopornpanish K , Jiraporncharoen W , Hashmi A , Dejkriengkraikul N and Angkurawaranon C (2019) Utilisation of electronic health records for public health in Asia: a review of success factors and potential challenges. BioMed Research International 2019, 9. 10.1155/2019/7341841 PMC6644215 31360723

[ref22] Dumbreck S , Flynn A , Nairn M , Wilson M , Treweek S , Mercer SW , Alderson P , Thompson A , Payne K and Guthrie B (2015) Drug-disease and drug-drug interactions: systematic examination of recommendations in 12 UK national clinical guidelines. BMJ (Clinical Research edition) 350, h949–h949. 10.1136/bmj.h949 PMC4356453 25762567

[ref24] Ferrari R (2015) Writing narrative style literature reviews. Medical Writing 24, 230–235.

[ref25] Foo KM , Sundram M and Legido-Quigley H (2020) Facilitators and barriers of managing patients with multiple chronic conditions in the community: a qualitative study. BMC Public Health 20, 273. 32106838 10.1186/s12889-020-8375-8 PMC7045577

[ref26] Fortin M , Lapointe L , Hudon C , Vanasse A , Ntetu AL and Maltais D (2004) Multimorbidity and quality of life in primary care: a systematic review. Health and Quality of Life Outcomes 2, 51. 15380021 10.1186/1477-7525-2-51 PMC526383

[ref27] Frandsen BR , Joynt KE , Rebitzer JB and Jha AK (2015) Care fragmentation, quality, and costs among chronically ill patients. American Journal of Managed Care 21, 355–362. 26167702

[ref28] Glass DP , Kanter MH , Jacobsen SJ and Minardi PM (2017) The impact of improving access to primary care. Journal of Evaluation in Clinical Practice 23, 1451–1458. 28984018 10.1111/jep.12821 PMC5765488

[ref29] Harris M , Dennis S and Pillay M (2013) Multimorbidity Negotiating priorities and making progress. Australian Family Physician 42, 850–854. 24324984

[ref30] Hogg W , Lemelin J , Dahrouge S , Liddy C , Armstrong CD , Legault F , Dalziel B and Zhang W (2009) Randomized controlled trial of anticipatory and preventive multidisciplinary team care: for complex patients in a community-based primary care setting. Canadian Family Physician 55, e76–e85. 20008582 PMC2793206

[ref31] Hogg W , Lemelin J , Moroz I , Soto E and Russell G (2008) Improving prevention in primary care: evaluating the sustainability of outreach facilitation. Canadian Family Physician 54, 712–720. 18474705 PMC2377224

[ref32] Howard-Thompson A , Farland MZ , Byrd DC , Airee A , Thomas J , Campbell J , Cassidy R , Morgan T and Suda KJ (2013) Pharmacist-physician collaboration for diabetes care: cardiovascular outcomes. Annals of Pharmacotherapy 47, 1471–1477. 24285763 10.1177/1060028013504738

[ref33] Howe A (2012) What’s special about medical generalism? The RCGP’s response to the independent commission on generalism. British Journal of General Practice 62, 342–343. 10.3399/bjgp12X652175 PMC3381242 22781968

[ref34] Hsieh HM , Gu SM , Shin SJ , Kao HY , Lin YC and Chiu HC (2015) Cost-effectiveness of a diabetes pay-for-performance program in diabetes patients with multiple chronic conditions. PLoS One 10, e0133163. 26173086 10.1371/journal.pone.0133163 PMC4501765

[ref35] Ingersoll KS and Cohen J (2008) The impact of medication regimen factors on adherence to chronic treatment: a review of literature. Journal of Behavioral Medicine 31, 213–224. 18202907 10.1007/s10865-007-9147-y PMC2868342

[ref36] Kanesarajah J , Waller M , Whitty JA and Mishra GD (2018) Multimorbidity and quality of life at mid-life: a systematic review of general population studies. Maturitas 109, 53–62. 29452782 10.1016/j.maturitas.2017.12.004

[ref37] Katon WJ , Lin EH , Von Korff M , Ciechanowski P , Ludman EJ , Young B , Peterson D , Rutter CM , McGregor M and McCulloch D (2010) Collaborative care for patients with depression and chronic illnesses. New England Journal of Medicine 363, 2611–2620. 21190455 10.1056/NEJMoa1003955 PMC3312811

[ref38] Kernick D , Chew-Graham CA and O’Flynn N (2017) Clinical assessment and management of multimorbidity: NICE guideline. British Journal of General Practice 67, 235–236. 10.3399/bjgp17X690857 PMC5409424 28450343

[ref39] Köberlein-Neu J , Mennemann H , Hamacher S , Waltering I , Jaehde U , Schaffert C and Rose O (2016) Interprofessional medication management in patients with multiple morbidities. Deutsches Ärzteblatt International 113, 741–748. 27890050 10.3238/arztebl.2016.0741 PMC5159681

[ref40] Kodner D (2002) Integrated care: meaning, logic, applications, and implications-a discussion paper. International Journal of Integrated Care 2, 14. 10.5334/ijic.67 PMC1480401 16896389

[ref41] Kravet SJ , Shore AD , Miller R , Green GB , Kolodner K and Wright SM (2008) Health care utilization and the proportion of primary care physicians. American Journal of Medicine 121, 142–148. 18261503 10.1016/j.amjmed.2007.10.021

[ref42] Krska J , Cromarty JA , Arris F , Jamieson D , Hansford D , Duffus PR , Downie G and Seymour DG (2001) Pharmacist-led medication review in patients over 65: a randomized, controlled trial in primary care. Age Ageing 30, 205–211. 11443021 10.1093/ageing/30.3.205

[ref43] Leutz WN (1999) Five laws for integrating medical and social services: lessons from the United States and the United Kingdom. Milbank Quarterly 77, 77–110, iv–v. 10197028 10.1111/1468-0009.00125 PMC2751110

[ref44] Levine C , Reinhard SC , Feinberg LF , Albert S and Heart A (2003–2004) Family caregivers on teh job: moving beyond ADLs and IADLs. Generations: Journal of the American Society on Aging 27, 17–23.

[ref45] Lynch EB , Liebman R , Ventrelle J , Avery EF and Richardson D (2014) A self-management intervention for African Americans with comorbid diabetes and hypertension: a pilot randomized controlled trial. Preventing Chronic Disease 11, E90. 24874782 10.5888/pcd11.130349 PMC4040140

[ref46] Marengoni A , Angleman S , Melis R , Mangialasche F , Karp A , Garmen A , Meinow B and Fratiglioni L (2011) Aging with multimorbidity: a systematic review of the literature. Ageing Research Reviews 10, 430–439. 21402176 10.1016/j.arr.2011.03.003

[ref47] Marengoni A and Onder G (2015) Guidelines, polypharmacy, and drug-drug interactions in patients with multimorbidity. BMJ: British Medical Journal 350, h1059. 25761379 10.1136/bmj.h1059

[ref48] May C , Montori VM and Mair FS (2009) We need minimally disruptive medicine. BMJ 339, b2803. 19671932 10.1136/bmj.b2803

[ref49] McGilton KS , Vellani S , Yeung L , Chishtie J , Commisso E , Ploeg J , Andrew MK , Ayala AP , Gray M , Morgan D , Chow AF , Parrott E , Stephens D , Hale L , Keatings M , Walker J , Wodchis WP , Dubé V , McElhaney J and Puts M (2018) Identifying and understanding the health and social care needs of older adults with multiple chronic conditions and their caregivers: a scoping review. BMC Geriatrics 18, 231. 30285641 10.1186/s12877-018-0925-x PMC6167839

[ref50] Mercer SW , Fitzpatrick B , Guthrie B , Fenwick E , Grieve E , Lawson K , Boyer N , McConnachie A , Lloyd SM , O’Brien R , Watt GC and Wyke S (2016) The CARE Plus study – a whole-system intervention to improve quality of life of primary care patients with multimorbidity in areas of high socioeconomic deprivation: exploratory cluster randomised controlled trial and cost-utility analysis. BMC Medicine 14, 88. 27328975 10.1186/s12916-016-0634-2 PMC4916534

[ref52] Mitty E (2010) Iatrogenesis, frailty, and geriatric syndromes. Geriatric Nursing 31, 368–374. 20832910 10.1016/j.gerinurse.2010.08.004

[ref53] Moffat K and Mercer SW (2015) Challenges of managing people with multimorbidity in today’s healthcare systems. BMC Family Practice 16, 129. 26462820 10.1186/s12875-015-0344-4 PMC4604728

[ref54] Molokhia M and Majeed A (2017) Current and future perspectives on the management of polypharmacy. BMC Family Practice 18, 70. 28587644 10.1186/s12875-017-0642-0 PMC5461681

[ref55] Muth C , Kirchner H , van den Akker M , Scherer M and Glasziou PP (2014a) Current guidelines poorly address multimorbidity: pilot of the interaction matrix method. Journal of Clinical Epidemiology 67, 1242–1250. 25216898 10.1016/j.jclinepi.2014.07.004

[ref56] Muth C , van den Akker M , Blom JW , Mallen CD , Rochon J , Schellevis FG , Becker A , Beyer M , Gensichen J , Kirchner H , Perera R , Prados-Torres A , Scherer M , Thiem U , van den Bussche H and Glasziou PP (2014b) The Ariadne principles: how to handle multimorbidity in primary care consultations. BMC Medicine 12, 223. 25484244 10.1186/s12916-014-0223-1 PMC4259090

[ref57] Naganathan GKK , Gill A , Jaakkimainen L , Upshur R and Wodchis WP (2016) Perceived value of support for older adults coping with multi-morbidity: patient, informal care-giver and family physician perspectives. Ageing Society 36, 1891–1914.

[ref58] North S (2020) Telemedicine in the Time of COVID and Beyond. The Journal of Adolescent Health: Official Publication of the Society for Adolescent Medicine 67, 145–146. 32605827 10.1016/j.jadohealth.2020.05.024 PMC7320687

[ref59] Nunes BP , Flores TR , Mielke GI , Thumé E and Facchini LA (2016) Multimorbidity and mortality in older adults: a systematic review and meta-analysis. Archives of Gerontology and Geriatrics 67, 130–138. 27500661 10.1016/j.archger.2016.07.008

[ref60] Okeowo D , Patterson A , Boyd C , Reeve E , Gnjidic D and Todd A (2018) Clinical practice guidelines for older people with multimorbidity and life-limiting illness: what are the implications for deprescribing? Therapeutic Advances in Drug Safety 9, 619–630. 30479737 10.1177/2042098618795770 PMC6243426

[ref61] Omboni S , Campolo L and Panzeri E (2020) Telehealth in chronic disease management and the role of the Internet-of-Medical-Things: the Tholomeus® experience. Expert Review of Medical Devices 17, 659–670. 32536214 10.1080/17434440.2020.1782734

[ref62] Orozco-Beltran D , Sánchez-Molla M , Sanchez JJ , Mira JJ and Valcrònic Research G (2017) Telemedicine in primary care for patients with chronic conditions: the ValCrònic Quasi-experimental study. Journal of medical Internet Research 19, e400. 29246881 10.2196/jmir.7677 PMC5747596

[ref63] Palmer K , Marengoni A , Forjaz MJ , Jureviciene E , Laatikainen T , Mammarella F , Muth C , Navickas R , Prados-Torres A , Rijken M , Rothe U , Souchet L , Valderas J , Vontetsianos T , Zaletel J and Onder G (2018) Multimorbidity care model: recommendations from the consensus meeting of the Joint Action on Chronic Diseases and Promoting Healthy Ageing across the Life Cycle (JA-CHRODIS). Health Policy 122, 4–11. 28967492 10.1016/j.healthpol.2017.09.006

[ref64] Parsons PL , Slattum PW and Bleich M (2019) Mainstreaming health and wellness: the RHWP Innovation model to complement primary care. Nursing Forum 54, 263–269. 30693939 10.1111/nuf.12326 PMC6876661

[ref65] Pathirana TI and Jackson CA (2018) Socioeconomic status and multimorbidity: a systematic review and meta-analysis. Australian and New Zealand Journal of Public Health 42, 186–194. 29442409 10.1111/1753-6405.12762

[ref66] Plochg T and Klazinga NS (2002) Community-based integrated care: myth or must? International Journal for Quality in Health Care 14, 91–101. 11954688 10.1093/oxfordjournals.intqhc.a002606

[ref67] Poitras ME , Maltais ME , Bestard-Denommé L , Stewart M and Fortin M (2018) What are the effective elements in patient-centered and multimorbidity care? A scoping review. BMC Health Services Research 18, 446. 29898713 10.1186/s12913-018-3213-8 PMC6001147

[ref68] Prados-Torres A , Poblador-Plou B , Calderón-Larrañaga A , Gimeno-Feliu LA , González-Rubio F , Poncel-Falcó A , Sicras-Mainar A and Alcalá-Nalvaiz JT (2012) Multimorbidity patterns in primary care: interactions among chronic diseases using factor analysis. PLoS One 7, e32190. 22393389 10.1371/journal.pone.0032190 PMC3290548

[ref69] Rijken M , Hujala A , van Ginneken E , Melchiorre MG , Groenewegen P and Schellevis F (2018) Managing multimorbidity: profiles of integrated care approaches targeting people with multiple chronic conditions in Europe. Health Policy 122, 44–52. 29102089 10.1016/j.healthpol.2017.10.002

[ref101] Rijken M , Struckmann V , van der Heide I , Hujala A , Barbabella F , van Ginneken E and Schellevis F (2017) European observatory policy briefs. In Richardson E and van Ginneken E , editors, How to improve care for people with multimorbidity in Europe? Copenhagen, Denmark: European Observatory on Health Systems and Policies © NIVEL and TU Berlin 2017, 5–27. 29144712

[ref71] Rochon PA and Gurwitz JH (2017) The prescribing cascade revisited. Lancet 389, 1778–1780. 28495154 10.1016/S0140-6736(17)31188-1

[ref72] Salisbury C , Thomas C , Cathain A , Rogers A , Pope C , Yardley L , Hollinghurst S , Fahey T , Lewis G , Large S , Edwards L , Rowsell A , Segar J , Brownsell S and Montgomery AA (2015) TElehealth in CHronic disease: mixed-methods study to develop the TECH conceptual model for intervention design and evaluation. BMJ Open 5, e006448. 10.1136/bmjopen-2014-006448 PMC4322202 25659890

[ref73] Scott IA , Pillans PI , Barras M and Morris C (2018) Using EMR-enabled computerized decision support systems to reduce prescribing of potentially inappropriate medications: a narrative review. Therapeutic Advances in Drug Safety 9, 559–573. 30181862 10.1177/2042098618784809 PMC6116772

[ref74] Shi L (2012) The impact of primary care: a focused review. Scientifica (Cairo) 2012, 432892. 24278694 10.6064/2012/432892 PMC3820521

[ref75] Sinsky CA (2020) Implementing telemedicine in primary care: learning lessons from electronic health records. Mayo Clinic Proceedings 95, 1835–1837. 32861325 10.1016/j.mayocp.2020.07.017 PMC7449897

[ref76] Sommers LS , Marton KI , Barbaccia JC and Randolph J (2000) Physician, nurse, and social worker collaboration in primary care for chronically ill seniors. Archives of Internal Medicine 160, 1825–1833. 10871977 10.1001/archinte.160.12.1825

[ref77] Stein DJ , Benjet C , Gureje O , Lund C , Scott KM , Poznyak V and van Ommeren M (2019) Integrating mental health with other non-communicable diseases. BMJ 364, l295. 30692081 10.1136/bmj.l295 PMC6348425

[ref78] Suriyawongpaisal P , Aekplakorn W , Leerapan B , Lakha F , Srithamrongsawat S and von Bormann S (2019) Assessing system-based trainings for primary care teams and quality-of-life of patients with multimorbidity in Thailand: patient and provider surveys. BMC Family Practice 20, 85. 31208358 10.1186/s12875-019-0951-6 PMC6580542

[ref79] Susan Swientozielskyj SM , Palmer S , Palmer S , Kohn P , Daniel N , Carey L , Sevak L , Nightingale J , Kenwood J, Westwood J, Stamp J, Blower J, Murgatroyd H, Baggaley G, Bryant E, Cooper A , Nwosu A (2014) NHS England: multi-disciplinary team handbooks. NHS England/Nursing/LTC. https://www.england.nhs.uk/wp-content/uploads/2015/01/mdt-dev-guid-flat-fin.pdf.

[ref80] Tinetti ME , Bogardus ST , Joseph R and Agostini JV (2004) Potential pitfalls of disease-specific guidelines for patients with multiple conditions. New England Journal of Medicine 351, 2870–2874. 15625341 10.1056/NEJMsb042458

[ref81] Uhlig K , Leff B , Kent D , Dy S , Brunnhuber K , Burgers JS , Greenfield S , Guyatt G , High K , Leipzig R , Mulrow C , Schmader K , Schunemann H , Walter LC , Woodcock J and Boyd CM (2014) A framework for crafting clinical practice guidelines that are relevant to the care and management of people with multimorbidity. Journal of General Internal Medicine 29, 670–679. 24442332 10.1007/s11606-013-2659-y PMC3965742

[ref82] Uijen AA and van de Lisdonk EH (2008) Multimorbidity in primary care: prevalence and trend over the last 20 years. Journal of General Internal Medicine 14 (Suppl 1), 28–32. 10.1080/13814780802436093 18949641

[ref83] van den Bussche H , Schön G , Kolonko T , Hansen H , Wegscheider K , Glaeske G and Koller D (2011) Patterns of ambulatory medical care utilization in elderly patients with special reference to chronic diseases and multimorbidity—results from a claims data based observational study in Germany. BMC Geriatrics 11, 54. 21914191 10.1186/1471-2318-11-54 PMC3180370

[ref84] van Oostrom SH , Picavet HS , de Bruin SR , Stirbu I , Korevaar JC , Schellevis FG and Baan C A (2014) Multimorbidity of chronic diseases and health care utilization in general practice. BMC Family Practice 15, 61. 24708798 10.1186/1471-2296-15-61 PMC4021063

[ref85] Vishal Ahuja CA and Staats BR (2020) Maintaining continuity in service: an empirical examination of primary care physicians. Manufacturing & Service Operations Management. 22(5), 1–19.

[ref86] Wallace E , Salisbury C , Guthrie B , Lewis C , Fahey T and Smith SM (2015) Managing patients with multimorbidity in primary care. BMJ 350, h176. 25646760 10.1136/bmj.h176

[ref87] Willadsen TG , Siersma V , Nicolaisdóttir DR , Køster-Rasmussen R , Jarbøl,DE , Reventlow S , Mercer SW and Olivarius NF (2018) Multimorbidity and mortality: a 15-year longitudinal registry-based nationwide Danish population study. Journal of Comorbidity 8, 2235042x18804063. 10.1177/2235042X18804063 PMC6194940 30364387

[ref88] World Health Organization (2013) Arguing for universal coverage. Geneva: World Health Organization. https://apps.who.int/iris/handle/10665/204355.

[ref89] World Health Organization (2016) Multimorbidity. Technical series on safer primary care. Geneva: World Health Organization; 2016.

[ref90] Zulman DM , Jenchura EC , Cohen DM , Lewis ET , Houston TK , Asch SM (2015) How can eHealth technology address challenges related to multimorbidity? Perspectives from patients with multiple chronic conditions. Journal of General Internal Medicine 30, 1063–1070. 25691239 10.1007/s11606-015-3222-9 PMC4510242

