# Supplementary material for: Advancing multimorbidity management in primary care: a narrative review
Source: Prim Health Care Res Dev. 2022 Jul 1;23:e36. doi: 10.1017/S1463423622000238 (PMC9309754; doi:10.1017/S1463423622000238)
Supplement: Supplementary file 1 [file S1463423622000238sup001.docx]

**Supplementary Table 1: Evidence for system-based approach**

| **Author** | **Title** | **Population** | **Intervention** | **Outcome** | **Result** |
| --- | --- | --- | --- | --- | --- |
| 1. Karen Barnett 2012 | Epidemiology of multimorbidity and implications for health care, research, and medical education: a cross-sectional study | General population | - | The number of people with multimorbidity is stratified by disorder type, age, sex, and socioeconomic status | The number of people with multimorbidity increases with age and degree of socioeconomic deprivation. Mental disorders are prevalent in people with multimorbidity. |
| 2. Andrew Wister 2020 | Multimorbidity and Socioeconomic Deprivation among Older Adults: A Cross-sectional Analysis in Five Canadian Cities Using the CLSA | Older adults | Exposure: neighborhood-level socioeconomic deprivation | Ratio of multimorbidity | neighborhood-level socioeconomic deprivation was associated with multimorbidity |
| 3. Caroline Bähler 2015 | Multimorbidity, health care utilization and costs in an elderly community-dwelling population: a claims data based observational study | Patients aged 65 years old or older | Exposure: multimorbid | Health care utilization and costs | The number of consultations per year was 15.7 in patients with multimorbid vs. 4.4 consultations per year in patients with non-multimorbid.  Patients with multimorbid were 5.6 times more likely to be hospitalized.  Total costs were 5.5 times higher in multimorbid compared to non-multimorbid. |
| 4. Kah Mun Foo 2020 | Facilitators and barriers of managing patients with multiple chronic conditions in the community: a qualitative study | - | - | Describe facilitators and barriers of managing patients with multiple chronic conditions in the community in Singapore. | Some patients encountered financial difficulties in managing care. There is an inadequacy of the nationwide medical saving scheme to cover outpatient treatment and medications. Patients reported long consultation waiting times. Physicians were able to propose and drive quality improvement projects. There were challenges to delivering safe and quality care due to high patients load and waiting time, inadequate communication with specialists, and resource constraints.  Patients’ self-management styles are influenced by multiple factors, including work requirements, beliefs, and environment. |
| 5. David P. Glass 2017 | The impact of improving access to primary care | General population | Primary care at the worksite office, guaranteed same-day access, and no co-pay. | Health care utilization and costs | Higher primary care visit rate but lower urgent care visit rate in the intervention group. Higher cost in the intervention group. Potential for long-term costs reduction in the intervention group compared to the control group. |
| 6. Hui-Min Hsieh 2015 | Cost-Effectiveness of a Diabetes Pay-For-Performance Program in Diabetes Patients with Multiple Chronic Conditions | Patients with diabetes, with and without comorbid | Pay for performance (P4P) policy | Incremental cost-effectiveness ratios (ICERs) | QALYs for P4P patients and non-P4P patients were 2.80 and 2.71 for the DM alone cohort and 2.74 and 2.66 for the DM with comorbid cohort. The average incremental intervention costs per QALY in the DM alone cohort were TWD$167,251 and TWD$145,474 in DM with comorbid. |
| 7. Brigham R. Frandsen 2015 | Care Fragmentation, Quality, and Costs Among Chronically Ill Patients | Chronically ill patients | Exposure: high degree of fragmentation | Care quality and costs of care | Patients in the highest quartile of fragmentation, compared to the lowest quartile of fragmentation, overall had poorer care quality, a higher rate of preventable hospitalizations, and higher health care spending. |
| 8. Julie P.W. Bynum 2011 | Fewer Hospitalizations Result When Primary Care Is Highly Integrated into a Continuing Care Retirement Community | Older adults | Care model 1: A site that provides a wide range of health care services and provides all after-hours coverage. | Health care utilization | A higher hospitalizations rate was observed in Care model 2. The mortality rate was also higher in Care model 2. |
| 9. Steven J. Kravet 2008 | Health Care Utilization and the Proportion of Primary Care Physicians | Health care system | Contain primary care physicians | Association of health care utilization and proportion of primary care physicians to total physicians | Higher proportions of primary care physicians were associated with a decrease in health care utilization. Each 1% increase in the proportion was associated with a decrease in yearly utilization of 503 admissions. |
| 10. Paibul Suriyawongpaisal 2019 | Assessing system-based training for primary care teams and quality-of-life of patients with multimorbidity in Thailand: patient and provider surveys | Multimorbid patients | Different system-based training programs trained to health providers | Quality of life of the patients | Training programmes, which put more emphasis on bundling of competencies and contextualizing of applying such competencies, were positively associated with better quality of life. |
| 11. Vishal Ahuja 2018 | Maintaining Continuity in Service: An Empirical Examination of Primary Care Physicians | Diabetes patients | Different levels of continuity of care | Inpatient visits, length of stay, and readmission rate | Continuity of care is related to improvement in all three health outcomes. |
| 12. Katrin Uhlig 2014 | A Framework for Crafting Clinical Practice Guidelines that are Relevant to the Care and Management of People with Multimorbidity | - | - | Framework for crafting clinical practice guidelines that account for multimorbid | Multimorbidity is considered in almost every step of clinical practice guideline generation |
| 13. Daniel Okeowo 2018 | Clinical practice guidelines for older people with multimorbidity and life-limiting illness: what are the implications for deprescribing? | Older people with multimorbidity and life-limiting illness | NICE clinical guidelines | Explore the treatment regimen generated by the guidelines | As hypothetical patients have more health conditions, the number of medications increases dramatically. There are no discussed approaches to stopping the medication. |
| 14. Siobhan Dumbreck 2015 | Drug-disease and drug-drug interactions: systematic examination of recommendations in 12 UK national clinical guidelines | People with multiple chronic conditions with index condition | 12 NICE clinical guidelines on heart failure, type 2 diabetes, depression, atrial fibrillation, osteoarthritis, COPD, hypertension, post-myocardial infarction, dementia, rheumatoid arthritis, CKD, and neuropathic pain. | Frequency of drug interactions between guidelines. | There are multiple drug interactions between guidelines. |
| 15. Salisbury, C., 2018 | Management of multimorbidity using a patient-centred care model: a pragmatic cluster-randomised trial of the 3D approach | Adults with ≥ 3 chronic conditions | Six months:  Comprehensive 3D reviews incorporating patient-centered strategies VS usual care |  | There was no difference between intervention and control groups |
| 16. Alexandra Prados-Torres 2012 | Multimorbidity Patterns in Primary Care: Interactions among Chronic Diseases Using Factor Analysis | Patients with chronic disease multimorbidity | - | Multimorbidity patterns in primary care. | Multimorbidity was found in all age groups. 5 patterns of multimorbidity were identified: cardio-metabolic, psychiatric-substance abuse, mechanical-obesity-thyroidal, psychogeriatric and depressive. Some of these patterns were found to associate with age and sex. |

**Supplementary Table 2: Evidence for patient-centered approach**

| **Author** | **Title** | **population** | **intervention** | **Outcome measurement** | **result** |
| --- | --- | --- | --- | --- | --- |
| 1. Angkurawaranon 2020 | Process evaluation protocol of a cluster randomized trial for a scalable solution for delivery of Diabetes Self-Management Education in Thailand (DSME-T) | Patients with diabetes mellitus | **12 months:**  - DSME intervention | - change in HbA1C  - change in cardiovascular risk score | (protocol study) |
| 2. Mercer 2016 | The CARE Plus study - a whole-system intervention to improve quality of life of primary care patients with multimorbidity in areas of high socioeconomic deprivation: an exploratory cluster randomized controlled trial and cost-utility analysis | aged between 30 and 65 years and had two or more long-term conditions. | **12-month:**  - structured longer consultations - relationship continuity - practitioner support - self-management support | - quality of life (EQ-5D-5L utility scores)  - well-being (W-BQ12; 3 domains) - Cost-effectiveness | - ↑ negative well-being  - ↑ quality of life |
| 3. Boult 2011 | The effect of guided care teams on the use of health services: results from a cluster-randomized controlled trial. | Patients who were ≥65 years and at high risk of using health services heavily during the following period | **18 months:**  -“Guide Care” program (home assessment, individual management plan, self-management coach) VS usual care | - Health services use hospital admissions  - nursing facility use, visits, home health care  - quality of chronic care (PACIC score) | Intervention group:  - a higher 30-day hospital readmission  - lower hospital day  - lower ER visit  - higher specialty care visit  - lower PACIC score |
| 4. Hogg 2008 | Improving prevention in primary care: evaluating the sustainability of outreach facilitation. | Population of primary care practices | **15 months:**  - enhanced multidisciplinary team with structured home visit  - drug review  - patient care plan | % Appropriated management in each grade | Significant increase inappropriate delivery and reduction in inappropriate |
| 5. Katon 2010 | Collaborative care for patients with depression and chronic illnesses | Depression+DM/CAD | **12 months:**  - team care nurse  - structured visited  - patient care plan  - treatment target  - weekly team meeting  - electronic registry  - education of nurse manager  - patient self-care | - depression score (SCL-20)  - HbA1C  - BPs  - Cholesterol  - health-related QoL | Intervention group:  - lower depression  - lower HbA1C  - no significant differences in blood pressure  - lower cholesterol  - better health-related quality of life |
| 6. Krska 2001 | Pharmacist-led medication review in patients over 65: a randomized, controlled trial in primary care | Patients aged ≥ 65 years with ≥ 2 chronic conditions and having ≥ 4 medical prescriptions | **Three months:**  - patient care plan created by pharmacists + others | - pharmaceutical care issue | Intervention group:  - issue resolved better than the control group |
| 7. Sommers 2000 | Physician, nurse, and social worker collaboration in primary care for chronically ill seniors | Patients aged ≥ 65 years having ≥ 2 chronic conditions | **Two years:**  - enhanced social worker, home assessment, patient care plan  - training in care coordination | - health service  - patient health status |  |
| 8. Barley 2014 | The UPBEAT nurse-delivered  personalized care intervention for people with coronary heart disease who report current chest pain and depression:  A randomised controlled pilot study. | CAD+depression | **Six months:**  - UPBEAT intervention (nurse case manager, F/U by telephone call) | - depression (HADS-D and PHQ)  - chest pain | - no sig diff in depression  - |
| 9. Bogner 2008 | Integration of depression and hypertension treatment: a pilot, randomized controlled trial | HT+depression | **Six weeks:**  - integration of depression and HT treatment by integrated care managers | - depression score (CES-D)  - BP  - drug adherent | Intervention group:  - lower BP  - lower depression  - |
| 10. Lynch 2014 | A self-management intervention for African Americans with  comorbid diabetes and hypertension: a pilot randomized  controlled trial | DM+HT | **Six months:**  - DM self-management  - nutrition education  - behavior skills training  - self-monitoring skills  - goal-setting skills  - problem-solving skills | - weight loss  - HbA1C  -BP | -  - no significant. differences in blood pressure and HbA1C |
| 11. Howard Thompson A, et al. 2013 | Pharmacist-physician collaboration for diabetes care: cardiovascular outcomes | CV risk patients with DM | **1- to 12-wk intervals:**  - Pharmacists educated patients, reviewed blood glucose logs, ordered and monitored labs, and adjusted medications | Pre- vs. post-PPCM  - Reduction in SBP, DBP, and LDL-C  - Percentage of patients achieving BP goal (<130/80)  - Percentage of patients achieving LDL-C goal (<100mg/dl) | PPCM has a positive impact on CV risk in patients with T2DM. |
| 12. Hogg 2009 | Randomized controlled trial of Anticipatory and Preventive Multidisciplinary Team Care For complex patients in a community-based primary care setting |  | **14.4 months:**  Anticipatory and Preventive Team Care (APTCare) from a collaborative team composed of  their physicians, 1 of 3 nurse practitioners, and a pharmacist  VS usual care (FM alone) | - chronic disease management score (CDM) for diabetes  - preventive care | Intervention group;  - not significantly associated with the QOC score  - Improved preventive care by 16.5% |
| 13. Köberlein-Neu, J. 2016 | Interprofessional Medication Management in Patients With Multiple Morbidities | ≥ 2 chronic conditions in different organs + 1 CV disease | **15 months:**  1. medication management, and  2. care provided by the Pflege- und Wohnberatung (PuW, home-care specialists) | - quality of pharmacotherapy (MAI) | Intervention phase:  - significant reduction of mean MAI score |
| 14. Naganathan G, 2016 | Perceived value of support for older adults coping with multi-morbidity: patient, informal caregiver and family physician perspectives | Patients aged ≥ 65 years with ≥ 2 conditions | Semi-structural interview about care experiences, perception of support, goals of care, and frustrations | - | Formal care was considered positive by caregivers’ and providers’ views, but it was rejected by patients. Patients preferred informal care from their familiar caregivers. |
| 15. Christiane Muth 2014 | Current guidelines poorly address multimorbidity: pilot of the interaction matrix method | Patients with chronic heart failure (CHF) with other comorbidities | CHF guideline and other 47 guidelines | - | Propose a framework for identifying interactions between guidelines. Identify interactions between guidelines |
| 16. Zulman DM, 2015 | How can eHealth technology address challenges related to multimorbidity? Perspectives from patients with multiple chronic conditions. | Patients with ≥3 chronic conditions and experience using technology for health-related purposes | Focused group interview about using health technology | The purposes of technology use | 96% - health information  92% - communication with health providers  83% - track medical information  77% - track medications  55% - decision-making support regarding treatment |
